# *Afrostyrax lepidophyllus* Mildbr. and *Monodora myristica* (Gaertn.) Dunal Extracts Decrease Doxorubicin Cytotoxicity on H9c2 Cardiomyoblasts

**DOI:** 10.1155/2021/8858165

**Published:** 2021-02-22

**Authors:** Bruno M. Moukette, José P. Castelão-Baptista, Luciana Ferreira, Ana M. Silva, Rui F. Simões, Célia Cabral, Constant A. Pieme, Jeanne Y. Ngogang, Vilma A. Sardão, Paulo J. Oliveira

**Affiliations:** ^1^Laboratory of Biochemistry, Department of Biochemistry and Physiological Sciences, Faculty of Medicine and Biomedical Sciences, University of Yaoundé I, PO Box 1364, Yaoundé, Cameroon; ^2^CNC-Center for Neuroscience and Cell Biology, University of Coimbra, UC Biotech Building, Lot 8A, Biocant Park, Cantanhede 3060-197, Portugal; ^3^Department of Life Sciences, Faculty of Sciences and Technology, University of Coimbra, Coimbra, Portugal; ^4^University of Coimbra, Center for Innovative Biomedicine and Biotechnology (CIBB), Coimbra 3000-548, Portugal; ^5^University of Coimbra, Coimbra Institute for Clinical and Biomedical Research (iCBR), Clinic Academic Center of Coimbra (CACC), Faculty of Medicine, Coimbra 3000-548, Portugal

## Abstract

**Materials and Methods:**

Bark extracts of these plants (1 and 25 *µ*g/mL) were added 3 hours before coincubating H9c2 cardiomyoblasts with Dox (0.5 and 1 *µ*M) for 24 hours more. We measured cell mass and metabolic viability, mitochondrial transmembrane potential, superoxide anion content, and activity-like of caspase-3 and caspase-9 following treatment with the extracts and/or Dox. Also, selenium and vitamin C contents were measured in the plant extracts.

**Results:**

The results confirmed that Dox treatment decreased cell mass, mitochondrial membrane potential and metabolic viability, increased mitochondrial superoxide anion, and stimulated caspase-3 and caspase-9-like activities. Pretreatment of the cells with the plant extracts significantly inhibited Dox cytotoxicity, with more significant results at the higher concentration. Measurements of selenium and vitamin C in the extracts revealed higher concentration of both when compared with other Cameroonian spices.

**Conclusion:**

Both extracts of *A. lepidophyllus* and *M. myristica* were effective against Dox-induced cytotoxicity, most likely due to their content in antioxidants.

## 1. Introduction

Doxorubicin (Dox) is an anthracycline antibiotic commonly used alone or in combination with other therapeutics to treat several solid and hematologic tumors in both adult and pediatric patients [1, 2]. Dox anticancer activity is based on the molecule's ability to cleave DNA, mainly derived from interference with topoisomerase II, and to the generation of reactive oxygen species (ROS), resulting in cancer cell death [3].

Despite being a potent anticancer drug, the clinical use of Dox is impaired by its significant cumulative dose-dependent cardiotoxicity resulting in acute or long-term ventricular dysfunction and heart failure [4, 5]. The full picture of the mechanisms underlying Dox cardiotoxicity is complex [6]. The primarily documented hypothesis attributes Dox cardiotoxicity to direct and indirect mitochondrial-mediated effects, including reactive oxygen species (ROS)overproduction in that organelle, which causes decreased ability to produce ATP and can lead to cardiomyocyte cell death [7]. Dox also accumulates in mitochondria where it forms a complex with cardiolipin. The disruption of the cardiolipin-protein interface causes an increase of superoxide radical production [8], associated with a disruption in mitochondrial chain electron conductance. The increased production of ROS in cardiac mitochondria coupled with the reduced expression/activity of mitochondrial superoxide dismutase (SOD2) and other antioxidant agents contributes to increased oxidative damage to different structures including membranes, nucleic acids, and lipids [9]. Topoisomerase II beta (TOP2*β*) inhibition is also involved in the cardiomyopathy caused by anthracyclines [10]. Animal studies showed that the interactions between Dox and TOP2*β* cause DNA breaks and inhibit mitochondrial biogenesis in cardiomyocytes [11, 12]. We have previously shown in H9c2 cardiomyoblasts that Dox causes increased oxidative stress, a disruption of the mitochondrial transmembrane electric potential, increased expression of p53 and its downstream proapoptotic modulator Bax, and caspase-dependent and independent H9c2 cell death [1, 13].

Antioxidants have been used *in vivo* and *in vitro* to counteract Dox cardiotoxicity, although with mixed results [14–16]. Plant extracts contain antioxidant molecules, which are good candidates to prevent Dox cardiotoxicity [17]. Phenolic compounds found in high concentration in dietary plants are responsible for their antioxidant and cardioprotective effects [18, 19].

Extracts of plants used in traditional medicine, often validated over thousands of years, are often ignored in terms of their potential therapeutic effect and not considered for drug development purposes [20, 21]. Although several plant extracts with various action mechanisms were tested against Dox-induced cardiotoxicity both *in vitro* and *in vivo* [22], there is still an active search for more potential protective extracts. Some of the prevention mechanisms include antioxidant, anti-inflammatory, antiapoptotic, or survival enhancing activity [14]. It is not surprising that a great deal of attention is given to phytochemicals as an alternative to synthetic drugs, due to their reduced price and more extensive availability [23].

*Monodora myristica* (Gaertn.) Dunal (*M. myristica*), commonly known as “African nutmeg,” belongs to the family Annonaceae, and it is widely distributed in Central and West African countries. Its fruits are used as spices in various Cameroonian dishes [24]. Studies have shown that almost every part of *M. myristica* tree is important economically [25]. Specifically, bark is traditionally used to treat hemorrhoids, stomachache, fever pains, and eye diseases [26]. Previous screening of extracts from the bark and leaves revealed high content in rutin, eugenol, and quercetin [27].

*Afrostyrax lepidophyllus* Mildbr. (*A. lepidophyllus*), commonly known as “country onion,” belongs to the family Huaceae, and has a more restricted distribution, occurring in the tropical forest of some Central and West African countries [28]. Its fruits are used as a condiment in several African dishes [29]. The barks and roots of *A*. *lepidophyllus* are used in the treatment of urinary infections, indigestions, enema, female frigidity, and snake bites [30]. Although the chemical composition of seeds has been more studied, not much is known regarding the phytochemistry of the bark. A study by Moukette et al. reported a content of more than 28 mg/g of polyphenols for the hydroethanolic bark extract, of which around 7 mg/g were flavonoids [29]. The antiproliferative properties of the essential oils from the fruits of *A*. lepidophyllus against human glioblastoma (T98 G), human breast adenocarcinoma (MDA-MB 231), human malignant melanoma (A375), and human colon carcinoma (HCT116) cell lines have also been demonstrated [31]. The antioxidant and free radical scavenging and hepatoprotective properties of its leaves and barks were also reported in albino Wistar rats livers homogenates [29]. Still, despite both plants' good antioxidant properties, the effects of extracts from both *M. myristica* and *A*. lepidophyllus against Dox cytotoxicity in cardiac cells were never investigated.

In this work, our objective was to test the hypothesis that hydroethanolic bark extracts from both *M. myristica* and *A. lepidophyllus* decrease the cytotoxicity of Dox on H9c2 cardiomyoblasts, a cell line often used as a model for cardiac cells and previously used to investigate potential interventions against Dox cytotoxicity [32–36].

## 2. Materials and Methods

### 2.1. Reagents

Penicillin, streptomycin, fetal bovine serum (FBS), and trypsin were purchased from Gibco-Invitrogen (Grand Island, NY, USA). High-glucose Dulbecco's modified Eagle's medium (DMEM), doxorubicin, dithiothreitol (DTT), phenylmethanesulfonyl fluoride (PMSF), protease inhibitor cocktail (leupeptin, antipain, chymostatin, and pepstatin A), sulforhodamine B (SRB), and Bradford reagent were obtained from Sigma-Aldrich (St Quentin Fallavier, France). Tetramethylrhodamine-ethyl ester (TMRE) was purchased from Invitrogen (Paisley, UK). Nitric acid, perchloric acid, ethanol, hydrochloric acid, sodium borohydride, 2,4-dinitrophenylhydrazine, and sulfuric acid were purchased from Sigma-Aldrich (Hamburg, Germany). Dox was dissolved in Milli-Q water at a stock solution of 25 mM.

### 2.2. Plant Material

The barks of *A*. *lepidophyllus* and *M. myristica* were harvested in 2012 at Kala, a small village in the Center region of Cameroon. The plants were identified at the National Herbarium of Cameroon (YA) and voucher specimens were deposited under the numbers 39020 HNC and 27690/SFR/CAM, respectively. Both names were checked with Plant List (http://www.theplantslist.org). The samples were dried at room temperature, grounded into powder, and kept in dry conditions.

### 2.3. Water/Ethanol Extract Preparation

Powders were macerated at the ratio of 1:10 (w/v) for 48 hours in a mixture of water/ethanol (30/70; H_2_O pH = 3) to obtain the extract. The mixtures were then filtered using Buchner funnel and Whatman No.1 filter paper. The process was repeated after 24 hours. The supernatant was concentrated using a rotavapor and dried in a freeze-drier (SpeedVac concentrator, Labconco, Kansas City, MO, USA) for 24 hours. The dried powders were conditioned and kept at 4°C for further experiments. Before the experiments, both lyophilized powder extracts were solubilized in 30/70 water/ethanol in a 50 mg/mL stock solution.

### 2.4. Quantification of Selenium and Vitamin C

The concentrations of selenium and vitamin C, two important antioxidants [37, 38], were determined in the two extracts as described below.

#### 2.4.1. Determination of Selenium Content by Flame Atomic Absorptiometry

Determination of selenium (Se) content was performed as previously described by Diaz-Alarcon et al. [39]. This method consists of a mineralization of the samples and subsequent determination of Se content. A mass of 300 mg of the extract was weighed and mineralized by heating it with 5 mL of concentrated HNO_3_, at 80°C for 1 hour in a sand bath. Then, a volume of preparation in the ratio of 4 : l of HNO_3_ and HClO_4_ was added, and the heating process continued for an additional 3 hours until the sample was completely mineralized. Selenium content was measured after reducing Se^6+^ to Se^4+^ after 2 mL of concentrated HCl was added to the mineralized sample. The mixture was heated at 100°C for 10 minutes in a thermostated bath. Afterward, the sample was cooled and diluted with a solution of HCl 1.9%. An aliquot of this mixture was transferred to a reaction vessel, and the latter was placed in the MHS-10 system (Hydride generation system, Perkin Elmer, Waltham, MA, USA). After hydride generation using a NaBH_4_ solution, the absorbance measurement was completed in a quartz cuvette, heated over an air-acetylene flame at 196.0 nm.

#### 2.4.2. Determination of Vitamin C Content by Spectrophotometry

This assay is based on the oxidation of ascorbic acid to dehydroascorbic acid in the presence of a bromine solution. The L-dehydroascorbic acid formed then reacts with 2,4-DNPH to produce an osazone, which, when treated with 85% H_2_SO_4_, produces a red-colored complex [40]. A volume of 0.23 mL of bromine (3%) was added to 4 mL of diluted extract (100 *µ*g/mL). Then, 0.13 mL of thiourea (10%) was added to remove the excess of bromine. A volume of 1 mL of 2,4-DNPH was added to the mixture, and the preparation was incubated for 3 hours at 37°C in a thermostated bath. The sample was then allowed to cool in an ice bath for 30 minutes and treated with 5 mL of H_2_SO_4_ (85%), with constant stirring. The solution was allowed to stand for 30 minutes, and the absorbance was read at 521 nm. The results were expressed using a standard curve obtained with several dilutions of pure ascorbic acid.

### 2.5. Cell Culture and Treatment

The H9c2 cell line (CRL-1446™, ATCC, USA), initially isolated from embryogenic rat heart tissue after several passages [41], shares many similarities with primary cardiomyocytes, including membrane morphology, as well as molecular and physiological properties [42]. Therefore, the H9c2 cell line has been used to investigate Dox-induced cardiotoxicity by different groups including ours [1, 13, 43, 44]. Undifferentiated H9c2 cells were used as a model of a more youth heart, although differentiated cells share more common features to adult cardiomyocytes [32]. The cells were cultured in high-glucose Dulbecco's modified Eagle medium (DMEM) supplemented with 1.5 g/L sodium bicarbonate, 10% fetal bovine serum, 100 U/ml of penicillin, 0.25 *µ*g/mL of antimycotic (Fungizone® Gibco-Invitrogen), and 100 *µ*g/ml of streptomycin in 75 cm^2^ tissue culture flasks at 37°C in a humidified atmosphere of 5% CO_2_. The culture media were changed every two-three days, and cells were passaged once they attained approximately 80% of confluence to avoid cell fusion and formation of multinucleated myotubules [1]. Cells were seeded and 24 hours later, the medium was removed and replaced with fresh medium containing the plant extracts at the concentration of 1 *µ*g/mL or 25 *µ*g/mL in water/ethanol (30/70 v/v). As control, water/ethanol (30/70 v/v) (maximum of 0.05%) was used. The cells were then preincubated for 3 hours with the extracts before being coincubated for 24 hours with Dox at the concentrations of 0.5µM and 1µM.  .

### 2.6. Cell Viability Assays

#### 2.6.1. Sulforhodamine B (SRB) Assay

H9c2 cell mass was measured using sulforhodamine B (SRB) assay [45]. This test is based on the ability of SRB to bind to protein amino acid residues of fixed cells. The SRB sulfonic groups bind stoichiometrically to the essential amino acid residues in a slightly acidified medium, with the amount of SRB bonded to the cellular protein being directly proportional to the cell mass [46]. In order to assess the cytotoxic effect of the plant extracts in H9c2 cells and/or the effect on cellular proliferation, H9c2 cells were seeded in 48-well plates at the density of 35,000 cells per mL [1], and twenty-four hours after seeding, cells were treated with the plant extracts at the concentrations of 1 or 25 *µ*g/mL for 6, 24, or 48 hours. After treatment, the culture medium was removed, and the cells were rinsed with phosphate-buffered saline (PBS). Then, cells were then fixed in ice-cold methanol, supplemented with 1% acetic acid, and stored at −20°C for 24 hours. Following this process, the methanol/acetic acid was removed, and the plates were dried at 37°C. Later, 250 *µ*L of 0.05% of SRB in 1% acetic acid was added. The fixed cells were incubated with the SRB solution for 1 hour at 37°C. The SRB solution was then discarded, and the plates were rinsed with 1% acetic acid solution, to remove the excess of unbound SRB, dried at room temperature. A volume of 500 *µ*L of 10 mM Tris buffer (pH10) was added to dissolve the protein-bound SRB. The optical density was measured at 540 nm using a Biotek Cytation 3 reader (Biotek Instruments, Winooski, VT, USA). In order to assess the cytoprotection of the plant extracts against Dox-induced H9c2 cell death, H9c2 cells were pretreated with the plant extracts at the concentrations of 1 or 25 *µ*g/mL for 3 hours before Dox treatment. H9c2 cells were then treated with 0.5 or 1 *µ*M of Dox for 24 hours, as previously described [1, 47]. After Dox treatment, the culture medium was removed and H9c2 cells mass was measured as described above.

#### 2.6.2. Resazurin Reduction Test

The metabolic viability of H9c2 cells was assessed by the resazurin reduction assay [45]. This assay involves the reduction of the blue resazurin dye to a pink fluorescent compound (resorufin) by the respiratory metabolism of the living cells in the medium. The resazurin reduction is a direct measurement of the cellular metabolic activity [48]. Cells were treated with plant extracts and/or Dox as described in Section 2.6.1. After 24 hours of Dox exposure, the media were removed, and the cells were rinsed with 1% PBS and incubated with 150 *µ*L of culture medium supplemented with 10 *µ*g/mL of resazurin. Reduction to resorufin was measured by fluorimetry at 570 nm excitation and 600 nm emission using a Biotek Cytation 3 reader (Biotek Instruments, Winooski, VT, USA).

### 2.7. Measurement of the Mitochondrial Membrane Potential and Mitochondrial Superoxide Anion

#### 2.7.1. Mitochondrial Membrane Potential (Δ*ψ*m)

The Δ*ψ*m was indirectly assessed by staining H9c2 cells with the TMRE dye and its fluorescence determined in a microplate reader [49]. The assay is based on the ability of the positively charged TMRE (red-orange) to accumulate in the mitochondria of viable cells driven by the Δψm. Up to a certain dye concentration, hyperpolarized mitochondria will accumulate more cationic dye, while depolarized mitochondria will accumulate a lower amount [50]. This assay's cell treatment procedure was similar to the procedure used for the SRB and the resazurin assays [50]. The experimental procedure followed the protocol described by [49] with some modifications. After cell treatment, as described in Section 2.5, the medium was discarded, and the cells were rinsed with 200 *µ*L of basal medium. A volume of 100 *µ*L of TMRE (300 nM in HBSS medium) was added to the cells, which were incubated with the dye for 1 hour. The basal fluorescence was then read at 555 nm excitation wavelength and 580 nm emission wavelength using a Cytation 3 multiplate reader. Thereafter, 1 *µ*L of a solution of FCCP plus oligomycin (100 *µ*M/0.1 mg/mL) was added to the wells and the fluorescence was read at 555 nm excitation wavelength and 580 nm emission wavelength to obtain the maximum fluorescence. The cells were then fixed with ice-cold methanol in acetic acid, and the SRB assay was performed to normalize the fluorescence results to cell mass content.

#### 2.7.2. MitoSOX Fluorescent Assay

The effect of the plant extracts on mitochondrial superoxide anion was determined using the fluorescence dye[49] MitoSOX Red, a cationic derivative of dihydroethidium (DHE), which reacts with superoxide anion in the mitochondrial matrix [51]. MitoSOX Red is oxidized by mitochondrial superoxide anion to form 2-hydroxymitoethidium, which excites and emits at 510 and 580 nm, respectively, and exhibits red fluorescence [33]. This assay's cell handling procedure was similar to the ones used for the SRB and the resazurin assays (Section 2.6.1). After pretreatment with the extracts and treatment with Dox, the medium was removed, and the cells were washed twice with PBS. Then, 50 *µ*L of MitoSOX working solution (5 *µ*M in basal medium) was added to the cells, followed by an incubation with the dye solution for 20 minutes at 37°C. The fluorescence was read for 90 min, at 37°C, with 510 nm excitation wavelength and 580 nm emission wavelength using a Cytation 3 multiplate reader. The cells were then fixed with ice-cold methanol in acetic acid, and the SRB assay was performed to normalize the results.

### 2.8. Measurement of the Caspase-3 and Caspase-9-Like Activities

After treating H9c2 cells as previously described (Section 2.6.1), in 150-millimeter diameter dishes, the medium was removed, and cells were rinsed with PBS, and 200 *µ*L of lysis buffer (50 mM HEPES/NaOH, pH 7.4, 100 mM NaCl, 0.1% CHAPS, 10% glycerol) supplemented with 2 mM dithiothreitol (DTT) and protease inhibitor cocktail (containing 1 *µ*g/ml of leupeptin, antipain, chymostatin, and pepstatin A) was added [44]. The cells were harvested using a cell scraper and transferred into 1 mL centrifuge tubes. The samples were submitted to 3 cycles of freezing/thawing in a liquid nitrogen/water bath (37°C) while stirring the samples after each cycle. The cellular extracts were then passed (30 passages) through a 27-gauge needle. The lysed cells were centrifuged at 14,000 xg for 5 minutes (4°C), and the supernatant was collected and kept at −80°C until used. The protein contents were determined using the Bradford method, and bovine serum albumin was used as standard [44].

To measure caspase-9 and caspase-3-like activities, aliquots of cell extracts containing 50 *μ*g and 25 *μ*g of protein, respectively, were incubated in reaction buffer containing 25 mM HEPES (pH 7.5), 10% sucrose, 10 mM DTT, 0.1% CHAPS, and 100 *µ*M caspase substrates Ac-LEHD-pNA (caspase-9) or Ac-DEDV-pNA (caspase-3), for 2 hours at 37°C. Caspase-like activities were determined by following the colorimetric detection of the chromophore p-nitroanilide at 405 nm after cleavage from the labeled substrate. The method was calibrated with known concentrations of p-nitroanilide (pNA), and the results were expressed as % pNA released.

### 2.9. Statistical Analysis

Data statistical analyses were performed by using GraphPad Prism 6.0 program (GraphPad Software, Inc., La Jolla, CA, USA). Data is expressed as means ± SD, of six independent experiments, with 6 biological replicates for each condition. Multiple comparisons were performed using two-way analysis of variance (2-way ANOVA) followed by Dunnett's multiple comparisons test. Comparisons between two treatment groups were performed using a Student's *t*-test. Significance was accepted when *p* value < 0.05.

## 3. Results

### 3.1. Characterization of Selenium and Vitamin C Content in *A*. *lepidophyllus* and *M. myristica* Extracts

Selenium is an important micronutrient that has been reported to improve the cellular antioxidant network, including having a protective effect on cultured cardiomyocytes submitted to hypoxia/reoxygenation [52]. Our results demonstrated a high concentration of selenium in both plant extracts, respectively, 1.87 ± 0.51 mg/Kg for *A. lepidophyllus* and 1.05 ± 0.01 mg/Kg for *M. myristica* (Table 1), as compared to other Cameroonian spices such as *Echinops giganteus* (<0.5 ± 0.00 mg/Kg) and *Aframomum daniellii* (0.7 ± 0.03 mg/Kg) [53]. The concentration of vitamin C, a critical antioxidant nutrient [37], was determined in *A*. *lepidophyllus* (Afro) and *M. myristica* (Mono) extracts. Our results showed that their respective content in vitamin C (2.57 ± 0.87 mg/Kg for Afro and 2.89 ± 0.55 mg/Kg for Mono) was higher than that of several plants with similar properties (Table 1).

### 3.2. *A. lepidophyllus* and *M. myristica* Extracts Prevent Dox-Induced Decreased H9c2 Cells Mass and Metabolic Viability

**Figure 1:**
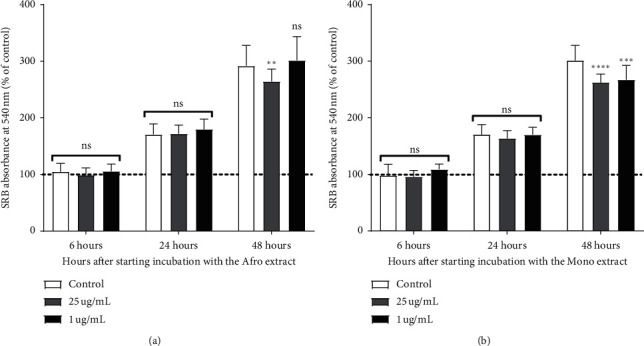
Effect of *Afrostyrax lepidophyllus* (a) and *Monodora myristica* (b) extracts on H9c2 growth rate, measured by the SRB assay. Cell treatment was performed as described in Section 2.5. The results represent mean ± SD of six independent experiments, with two biological replicates of each condition. ^*∗∗*^*p*=0.00082; ^*∗∗∗*^*p*=0.0004; ^*∗∗∗∗*^*p*=0.0001. Afro: *Afrostyrax lepidophyllus* water/ethanol extract. Mono: *Monodora myristica* water/ethanol extract. Control: H_2_O/ethanol (30/70).

**Figure 2:**
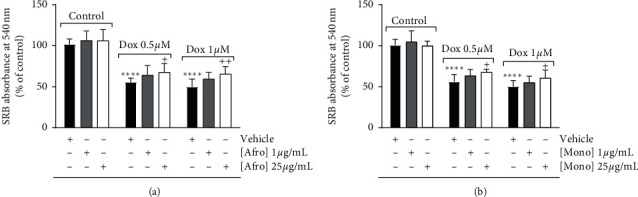
Dose-dependent effects of *Afrostyrax lepidophyllus* (a) and *Monodora myristica* (b) extracts on H9c2 cell mass measured by the SRB assay. Cell treatment was performed as described in Section 2.5. The results represent mean ± SD of six biological replicates. ^*∗∗∗∗*^*p* < 0.001 vs. control (no Dox added), ^+^*p* < 0.05 vs. Dox only; ^++^*p* < 0.01 vs. Dox (untreated cells treated with Dox). Dox: doxorubicin. Afro: *Afrostyrax lepidophyllus* water/ethanol extract. Mono: *Monodora myristica* water/ethanol extract. Vehicle: H_2_O/ethanol (30/70).

Before assessing the plant extracts' cytoprotection against Dox-induced H9c2 cell death, the cytotoxicity of the extracts and their effect on H9c2 proliferation were evaluated using two of the concentrations (1 and 25 *µ*g/mL) previously tested in a pilot dose-response study. The data shows that plant extracts, per se, did not interfere with cell mass after 24-hour treatment. At the 48-hour time point, a small, but significant decrease of cell mass was observed for both extracts (Figures 1(a) and 1(b)). H9c2 cardiomyoblasts were pretreated with the plant extracts for 3 hours before their incubation with Dox for an additional 24 hours. The results (Figures 2(a) and 2(b)) show a significant decrease of around 50% of cell mass caused by Dox treatment, for both 0.5 *µ*M and 1 *µ*M concentrations. Both cell extracts, at the concentration of 25 µg/mL, protected H9c2 cells against Dox cytotoxicity (Figures 2(a) and 2(b)) .

**Figure 3:**
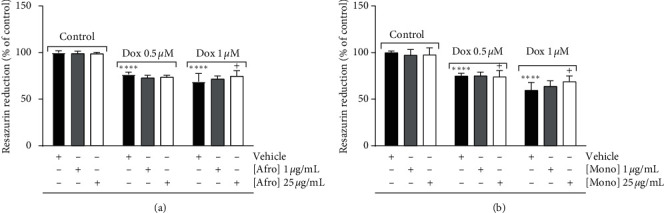
Dose-dependent effects of *Afrostyrax lepidophyllus* (a) and *Monodora myristica* (b) extracts on H9c2 cell metabolic viability measured using resazurin reduction. Cell treatment was performed as described in Section 2.5. The results represent mean ± SD of six biological replicates. ^*∗∗∗∗*^*p* < 0.001 vs. control (no Dox added), ^+^*p* < 0.05 vs. Dox only. Dox: doxorubicin. Afro: *Afrostyrax lepidophyllus* water/ethanol extract. Mono: *Monodora myristica* water/ethanol extract. Vehicle: H_2_O/ethanol (30/70).

The metabolic viability of H9c2 cardiomyoblasts was also analyzed using the resazurin assay. Our results confirmed that Dox, at both concentrations tested, decreased H9c2 metabolic viability, as it is clear from the data that the highest concentration of plant extract (25 *µ*g/mL) partly prevented the loss of cellular metabolic viability by around 6% (Afro) and 9% (Mono), but only in the highest concentration of Dox tested (1 *µ*M) (Figures 3(a) and 3(b)). As with the previous method, plant extracts, per se, did not show any cytotoxicity in terms of decreased metabolic viability.

### 3.3. *A. lepidophyllus* and *M. myristica* Extracts Prevent Dox-Induced Mitochondrial Membrane Potential (ΔΨm) Depolarization

**Figure 4:**
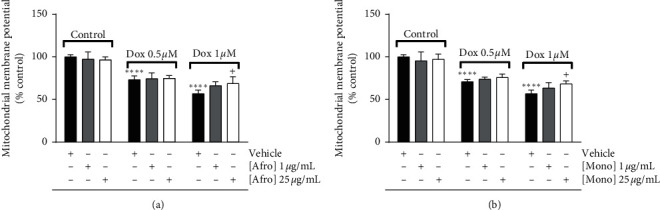
Dose-dependent effects of *Afrostyrax lepidophyllus* (a) and *Monodora myristica* (b) extracts on H9c2 mitochondrial membrane potential measured using TMRE staining. Cell treatment was performed as described in Section 2.5. The results represent the mean ± SD of six biological replicates. ^*∗∗∗∗*^*p* < 0.001 vs. control (no Dox added), ^+^*p* < 0.05 vs. Dox (untreated cells treated with Dox). Dox: doxorubicin. Afro: *Afrostyrax lepidophyllus* water/ethanol extract. Mono: *Monodora myristica* water/ethanol extract. Vehicle: H_2_O/ethanol (30/70).

The mitochondrial membrane potential (ΔΨm) is a critical mediator for ATP generation and for several mitochondrial roles in the cell [54]. A decrease of ΔΨm, caused by excessive oxidative stress, has been described to play a fundamental role in Dox-induced ΔΨm depletion [55]. The protective effects of the two plant extracts against Dox-induced H9c2 mitochondrial depolarization were determined using the mitochondrial specific TMRE dye, which accumulates in mitochondria based on the ΔΨm. In agreement with previous results, both concentrations of Dox led to ΔΨm depolarization, observed as a loss of TMRE fluorescence. Agreeing with the experimental endpoints, our results showed that the plant extracts, at 25 *µ*g/mL, prevented the decrease in ΔΨm after Dox treatment, although a statistically significant difference was only observed for 1 *µ*M Dox (Figures 4(a) and 4(b)).

### 3.4. *A. lepidophyllus* and *M. myristica* Extracts Decrease Dox-Induced Increased Mitochondrial Superoxide Anion in H9c2 Cardiomyoblasts

**Figure 5:**
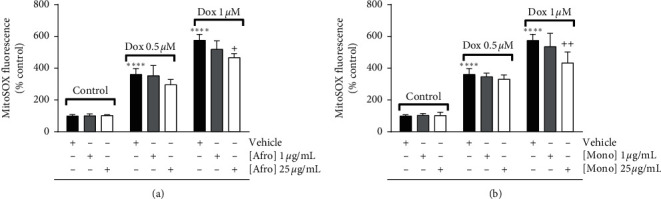
Dose-dependent effects of *Afrostyrax lepidophyllus* (a) and *Monodora myristica* (b) extracts on mitochondrial superoxide anion in H9c2 cells. Cell treatment was performed as described in Section 2.5. The results represent the mean ± SD of six biological replicates. ^*∗∗∗∗*^*p* < 0.001 vs. control (no Dox added), ^+^*p* < 0.05 vs. Dox; ^++^*p* < 0.01 vs. Dox (untreated cells treated with Dox). Dox: doxorubicin. Afro: *Afrostyrax lepidophyllus* water/ethanol extract. Mono: *Monodora myristica* water/ethanol extract. Vehicle: H_2_O/ethanol (30/70).

Increased generation of mitochondrial ROS in biological models in the context of Dox-induced cardiotoxicity was previously described [56], including in H9c2 cardiomyoblasts [1]. In agreement with this, the incubation of H9c2 cells with Dox in the present study resulted in a dose-dependent increase in MitoSOX fluorescence, evidence of increased reactivity with superoxide anion in mitochondria (Figure 5). Our results demonstrated a decrease of around 10 and 40% in MitoSOX fluorescence in H9c2 cells preincubated with *A. lepidophyllus* and *M. myristica* plant extracts, respectively, at 25 *µ*g/mL prior to Dox incubation (1 *µ*M) (Figures 5(a) and 5(b)).

### 3.5. *A. lepidophyllus* and *M. myristica* Extracts Decrease Caspase-3 and Caspase-9-Like Activities in H9c2 Cardiomyoblasts

**Figure 6:**
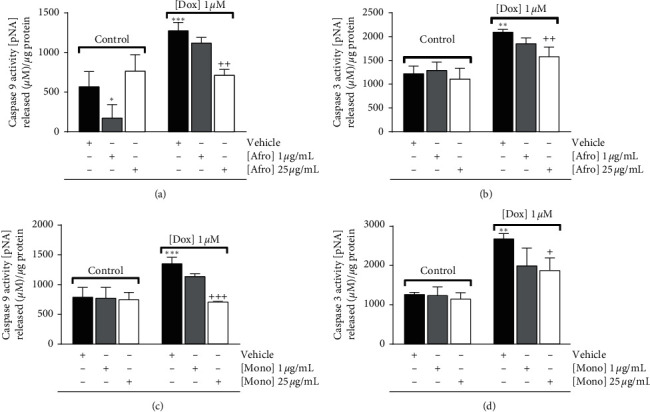
Dose-dependent effects of *Afrostyrax lepidophyllus* (a, b) and *Monodora myristica* (c, d) extracts on caspase-3 and caspase-9 activities. Cell treatment was performed as described in Section 2.5. The results represent mean ± SD of six biological replicates. ^*∗∗∗*^*p* < 0.01 vs. control; ^*∗∗*^*p* < 0.01 vs. control (no Dox added); ^*∗*^*p* < 0.05 vs. control (no Dox added); ^+^*p* < 0.05 vs. Dox; ^++^*p* < 0.01 vs. Dox; ^+++^*p* < 0.001 vs. Dox (untreated cells treated with Dox). Dox: doxorubicin. Afro: *Afrostyrax lepidophyllus* water/ethanol extract. Mono: *Monodora myristica* water/ethanol extract. Vehicle: H_2_O/ethanol (30/70).

Caspase-dependent cell death is implicated in the toxicity of Dox on H9c2 cells [44]. Our next aim was to investigate whether *A*. *lepidophyllus* and *M. myristica* could prevent the increase of caspase-like activity after Dox treatment (Figure 6). After treating the cells as described above (Section 2.5), caspase-3 and caspase-9-like activities were measured using a colorimetric method. As previously demonstrated [44], Dox treatment resulted in increased caspase-3 and caspase-9-like activities in H9c2 myoblasts. The results showed that *A*. *lepidophyllus* and *M. myristica* extracts at 25 *µ*g/mL decreased caspase-3 and caspase-9-like activities (by 517 and 560 units for Afro and 800 and 645 units for Mono, respectively) when cells were treated with 1 *µ*M Dox, as shown in Figures 6(a)–6(d).

## 4. Discussion

Doxorubicin is a highly active therapeutic agent used against a vast number of malignant diseases, including hematological and solid tumors [57]; however, the clinical application of Dox in cancer treatment is limited due to its cardiotoxic side effects. Among other effects, Dox leads to the generation of ROS that causes damage in cardiomyocytes, which may contribute to the development of cumulative cardiomyopathy, possibly resulting in heart failure [58]. It has been challenging to prevent the deleterious effects of Dox without affecting its pharmacological properties [58]. Therefore, the pursuit of an efficient and safe antagonist of Dox-induced cardiotoxicity remains a challenge. Several reports have focused on how phytochemicals, including polyphenols, may exert antioxidant protection through multiple pathways and their potential application in the management of Dox cardiotoxicity [57]. The present study investigated the potential protective effects of *A. lepidophyllus* and *M. myristica* extracts against Dox-induced cardiotoxicity in H9c2 cells. These two plants were chosen because of their uses in traditional Cameroonian medicine for the treatment of a variety of pathological conditions and because of previous publications reporting their high content in phenolic compounds [27, 29, 31, 59] and their potential anticancer activity [60, 61].

Under our experimental conditions, Dox (0.5 and 1 *μ*M for 24 hours) caused cytotoxicity on H9c2 cells, as indicated by cell mass loss, decreased metabolic viability, loss of ΔΨm, increased levels of mitochondrial superoxide anion, and increased caspase-3 and caspase-9-like activity, consistent with our previous studies in the same model [62, 63]. Our results demonstrated that pretreatment with both plant extracts (1 and 25 *µ*g/mL) prevented Dox-induced cytotoxicity in H9c2 cells, especially at the highest concentration tested. It is noteworthy to observe that the extracts, per se, showed no relevant toxicity, when cell mass was measured after 24 hours of incubation. As for the 48-hour treatment time point, a small decrease in cell mass was observed.

Diverse mechanisms are involved in Dox-induced apoptosis, including binding to mitochondrial membranes and pro-oxidant redox cycles [62]. Our results showed that pretreatment for 3 hours with the extracts from both plant promoted a decrease in Dox-induced mitochondrial superoxide anion and increased mitochondrial membrane potential H9c2 cells when compared to the Dox-treated group. These results are consistent with previously published reports which demonstrated the protective effects of other plant extracts on the mitochondrial membrane potential of H9c2 cells, as well as a reduction of Dox-induced ROS production [62]. Our results revealed a decrease in caspase-3 and caspase-9-like activities in cells pretreated for 3 hours with 25 *µ*g/mL of extracts before treatment with 1 *μ*M of Dox, as compared to the group treated with Dox alone, which predicts the ability of the two extracts to prevent Dox-induced cell apoptosis. Our findings are consistent with previous reports which indicated that natural products, such as berberine, inhibited Dox-induced apoptosis by disturbing ROS-induced p53 activation, mitochondrial dysfunction, executioner caspase activation, and activation of AMP-activated protein kinase (AMPK) [64].

One explanation for the results is that the plant extracts' antioxidant activity may be responsible for the observed protection against Dox cytotoxicity. We previously demonstrated the high phenolic content of these same extracts [27, 29]. Polyphenols, including flavonoids, may prevent Dox-induced cardiotoxicity through their free radical scavenging and antioxidant properties [62, 63].

These extracts are also endowed with a significant content of selenium and vitamin C, which can also contribute to reducing Dox-induced damage in the cells. In fact, selenium and vitamin C concentrations in the two extracts were higher than those found in other commonly used Cameroonian spices [53].

Selenium is a critical component of the antioxidant enzyme glutathione peroxidase (GPx) active site, playing an essential role in its spatial conformation and its catalytic activity [52]. The concentration of selenium in the surrounding environment has been correlated with the activity of the enzyme GPx and has significant consequences on the susceptibility of the cardiac tissue to oxidative stress [52]. Selenium content in the samples may increase the activity of GPx in H9c2 cells, thus, reducing Dox-induced oxidative stress. These results agree with previous reports demonstrating the protective effects of selenium supplementation in cultured cardiomyocytes submitted to hypoxia, and the inhibition of Dox-induced cardiotoxicity by preserving endogenous antioxidants [52].

Recent findings reported that vitamin C (25 *µ*M) decreased ROS generation and increased H9c2 viability after Dox treatment [65]. Vitamin C content in the extracts from *A. lepidophyllus* and *M. myristica* is higher than those found in *Mondia whitei* and *Aframomum daniellii* [53]. Therefore, in those extracts, vitamin C may protect cellular lipids against peroxidative damage by scavenging ROS in the aqueous phase before they can initiate lipid peroxidation [66]. Although a very limited analysis, Supplementary Figure 1 (Figure S1) shows the fold-protection for each extract in the maximum concentration tested. It must be stressed that other phytochemicals present in each extract may play an even more potent role in the protection against Dox cytotoxicity; however, the crude analysis in Figure S1 shows that a direct relationship between protection in most parameters analyzed and the total selenium/vitamin C (when considered isolated) was observed for vitamin C. Selenium and vitamin C content has been highlighted in this work because it sets *M. myristica* and *A. lepidophyllus* apart from the previously studied plants in this field.

When comparing the two extracts, *M. myristica* showed a higher protective effect against Dox than *A. lepidophyllus*. One possibility is that the higher content in vitamin C present in *M. myristica* could account for the difference, although both plants' phenolic composition and their intrinsic antioxidant activity may also explain at least some of the differences [29, 67].

In conclusion, the current study showed that extracts from *A. lepidophyllus* and *M. myristica* exerted protection against *in vitro* Dox cytotoxicity on H9c2 cells by decreasing mitochondrial superoxide anion, preserving mitochondrial membrane potential, and reducing caspase-like activity. Therefore, this initial study indicates that both extracts should be further investigated in animal models of Dox-induced cardiotoxicity to potentially validate a novel antioxidant therapeutic strategy to counteract Dox cardiotoxicity.

## Figures and Tables

**Table 1:** Total selenium and vitamin C contents in *Afrostyrax lepidophyllus* and *Monodora myristica* barks.

Extract	Se (mg/kg)	Vitamin C (mg/100 g)	Reference
*Afrostyrax lepidophyllus*	1.87 ± 0.51	2.57 ± 0.87	—
*Monodora myristica*	1.05 ± 0.01	2.89 ± 0.55	—
*Echinops giganteus*	<0.5 ± 0.00	1.26 ± 0.02	[53]
*Aframomum daniellii*	0.7 ± 0.03	0.94 ± 0.02	[53]
*Capsicum frutescens*	0.8 ± 0.04	1.42 ± 0.13	[53]
*Mondia whitei*	<0.5 ± 0.00	0.4 ± 0.14	[53]

The results are presented in mean ± SD of three replicates.

## Data Availability

The analyzed data to support the findings of this study are included in the article. The raw data used to support the findings of this study are available from the corresponding author upon request.

## Abbreviations

Δ*ψ*m: Mitochondrial membrane potential
Afro: *Afrostyrax lepidophyllus* Mildbr.
AMPK: 5′'-Adenosine monophosphate-activated protein kinase
ATP: Adenosine triphosphate
CHAPS: 3-[(3-Cholamidopropyl)dimethylammonio]-1-propanesulfonate hydrate
CO_2_: Carbon dioxide
DHE: dihydroethidium
DMEM: Dulbecco's modified Eagle's medium
DNA: Deoxyribonucleic acid
2,4-DNPH: 2,4-Dinitrophenylhydrazine
Dox: Doxorubicin
DTT: Dithiothreitol
FBS: Fetal bovine serum
FCCP: Carbonyl cyanide-4-(trifluoromethoxy)phenylhydrazone
GPx: Glutathione peroxidase
HBSS: Hank's balanced salt solution
HCl: Hydrochloric acid
HClO_4_: Perchloric acid
HEPES: 4-(2-Hydroxyethyl)-1-piperazineethanesulfonic acid
HNO_3_: Nitric acid
H_2_SO_4_: Sulfuric acid
Mono: *Monodora myristica* (Gaertn.) Dunal
NaBH_4_: Sodium borohydride
NaCl: Sodium chloride
NaOH: Sodium hydroxide
PBS: Phosphate-buffered saline
pNA: p-nitroanilide
PMSF: Phenylmethanesulfonyl fluoride
p53: Cellular tumor antigen
ROS: Reactive oxygen species
SD: Standard deviation
Se: Selenium
SOD2: Superoxide dismutase
SRB: Sulforhodamine B
TMRE: Tetramethylrhodamine, ethyl ester, perchlorate.

## Supplementary Materials

Supplementary Materials Supplementary Figure 1 shows the comparison of the protection provided by the extracts across the various assays against Dox cytotoxicity, highlighting the dose dependence of selenium and vitamin C contents.
