# Supplementary material for: Afrostyrax lepidophyllus Mildbr. and Monodora myristica (Gaertn.) Dunal Extracts Decrease Doxorubicin Cytotoxicity on H9c2 Cardiomyoblasts
Source: Evid Based Complement Alternat Med. 2021 Feb 22;2021:8858165. doi: 10.1155/2021/8858165 (PMC7920721; doi:10.1155/2021/8858165)
Supplement: Supplementary Materials — Supplementary Figure 1 shows the comparison of the protection provided by the extracts across the various assays against Dox cytotoxicity, highlighting the dose dependence of selenium and vitamin C contents. [file 8858165.f1.pdf]

Afrostyrax lepidophyllus Mildbr. and _Monodora myristica_ (Gaertn.) Dunal Extracts Decrease Doxorubicin Cytotoxicity on H9c2 Cardiomyoblasts

(Moukette et al.)

Supplementary material

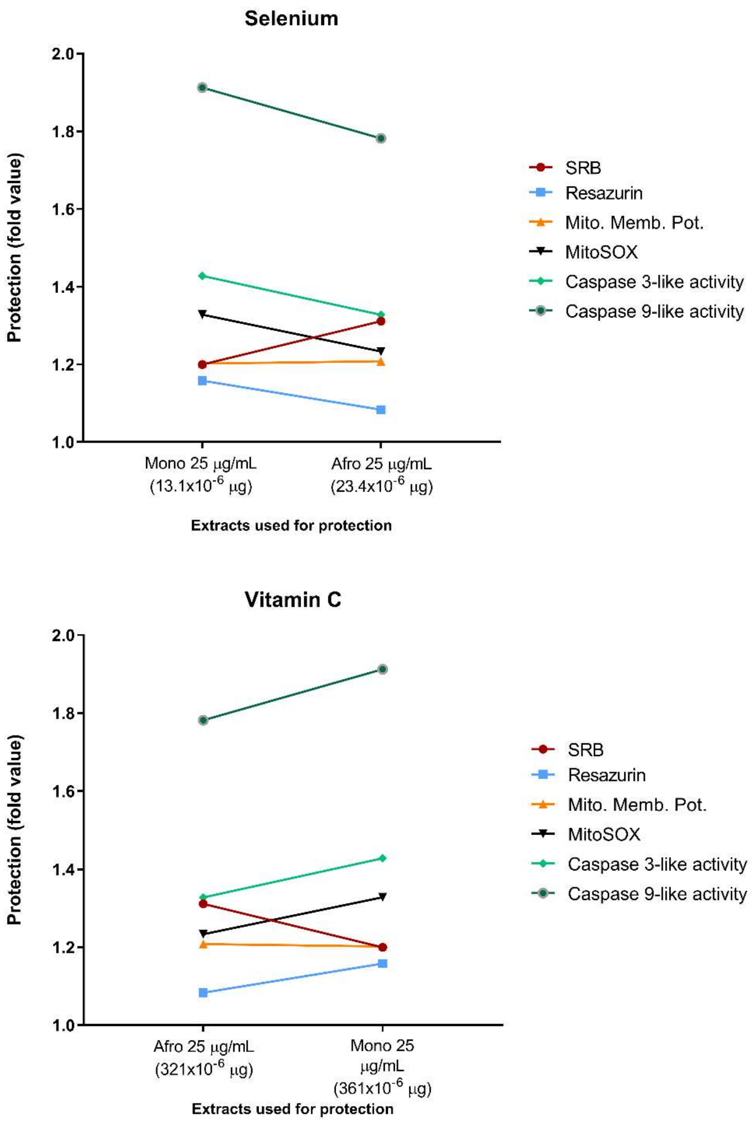

Supplementary Figure 1. Protection (fold value) of H9c2 against Dox comparison of the extracts across the various assays, highlighting the selenium and vitamin C contents of each extract.
